# Supplementary material for: Nigrostriatal dopamine modulates the striatal-amygdala pathway in auditory fear conditioning
Source: Nat Commun. 2023 Nov 9;14:7231. doi: 10.1038/s41467-023-43066-9 (PMC10636191; doi:10.1038/s41467-023-43066-9)
Supplement: Supplementary file 1 — Supplementary Information [file 41467_2023_43066_MOESM1_ESM.pdf]

Supplementary Figure 1

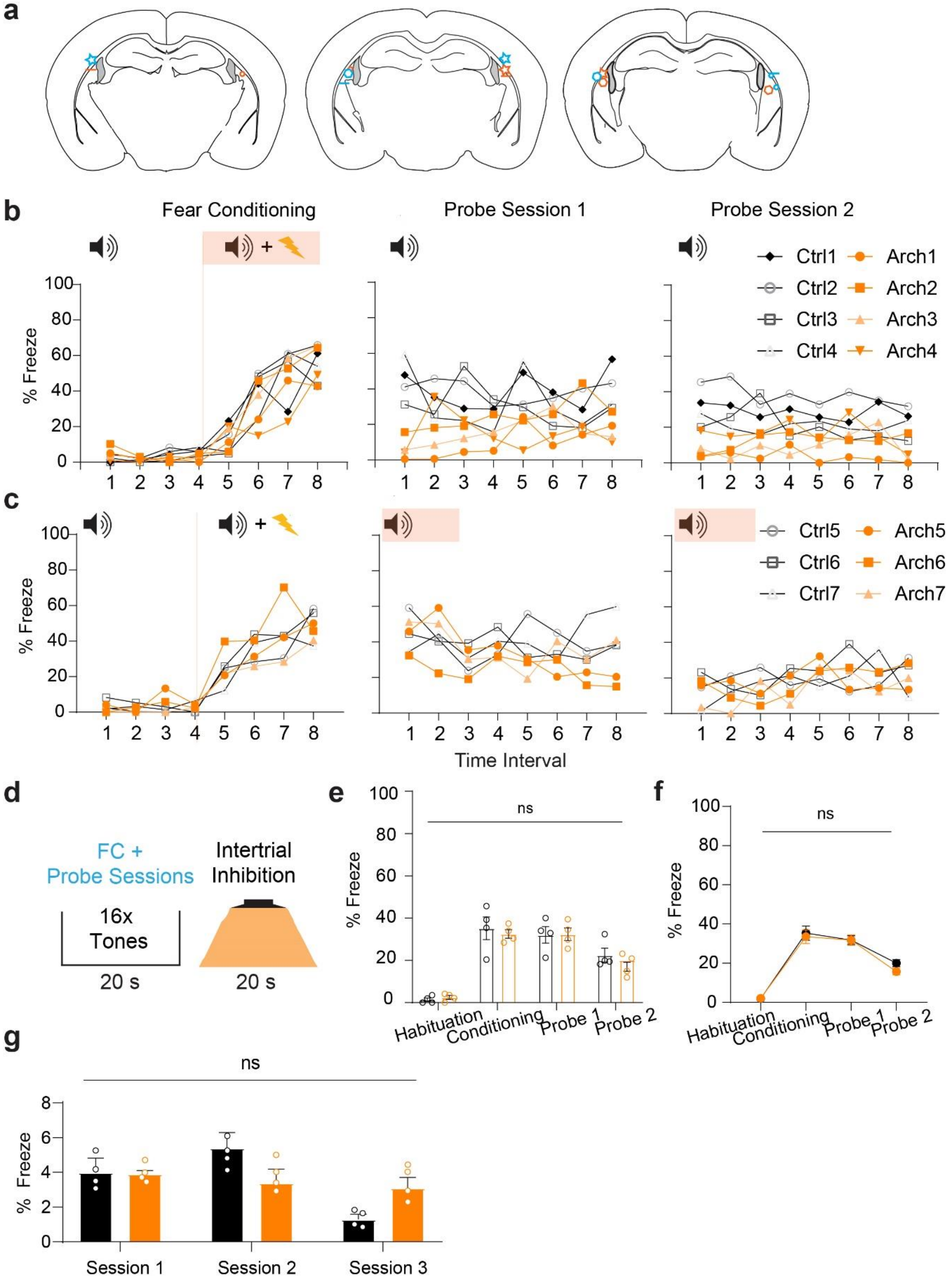

Supplementary Figure 1. Non-specific optogenetic silencing of the auditory striatal neuronal population does not impair fear memory formation or gross motor behavior. **a**, Summary of bilateral implantation sites for optogenetic experiments. Each distinct symbol represents an individual mouse. Cyan symbols, control GFP-only mice; orange symbols, ArchT mice. **b**, Conditioning tone-locked optogenetic silencing: freezing percentages for individual mice across conditioning and probe sessions for data summarized in **Fig. 1d-e**. **c**, Probe tone-locked optogenetic silencing: freezing percentages for individual mice across conditioning and probe sessions for data summarized in **Fig. 1f-g**. **d**, Schematic for intertrial optogenetic inhibition. Light was delivered for 20 s at random times outside of tone presentation during conditioning and probe sessions. **e**, Intertrial optogenetic silencing: bar plot of averaged freezing percentage in response to tones during habituation, conditioning, and probe sessions. Individual dots are averaged freezing percentages corresponding to individual animals. Error bars are SEM (two-sided unpaired Mann-Whitney test; ns, p = 0.37). **f**, Intertrial optogenetic silencing: line plot of averaged freezing percentage in response to tones during habituation, conditioning, and probe sessions. Error bars are SEM (two-sided unpaired Mann-Whitney test; ns, p =0.68). **g**, Freezing percentage during 30-min sessions in a spatial context distinct from that used for fear conditioning.

n = 4 mice per cohort. Black, GFP-control mice; orange, Arch mice. Error bars are SEM (unpaired Mann-Whitney test; ns for Session 1, p = 0.83; ns for Session 2, p = 0.29; ns for Session 3, p = 0.45). Source data are provided as a Source Data file.

Supplementary Figure 2

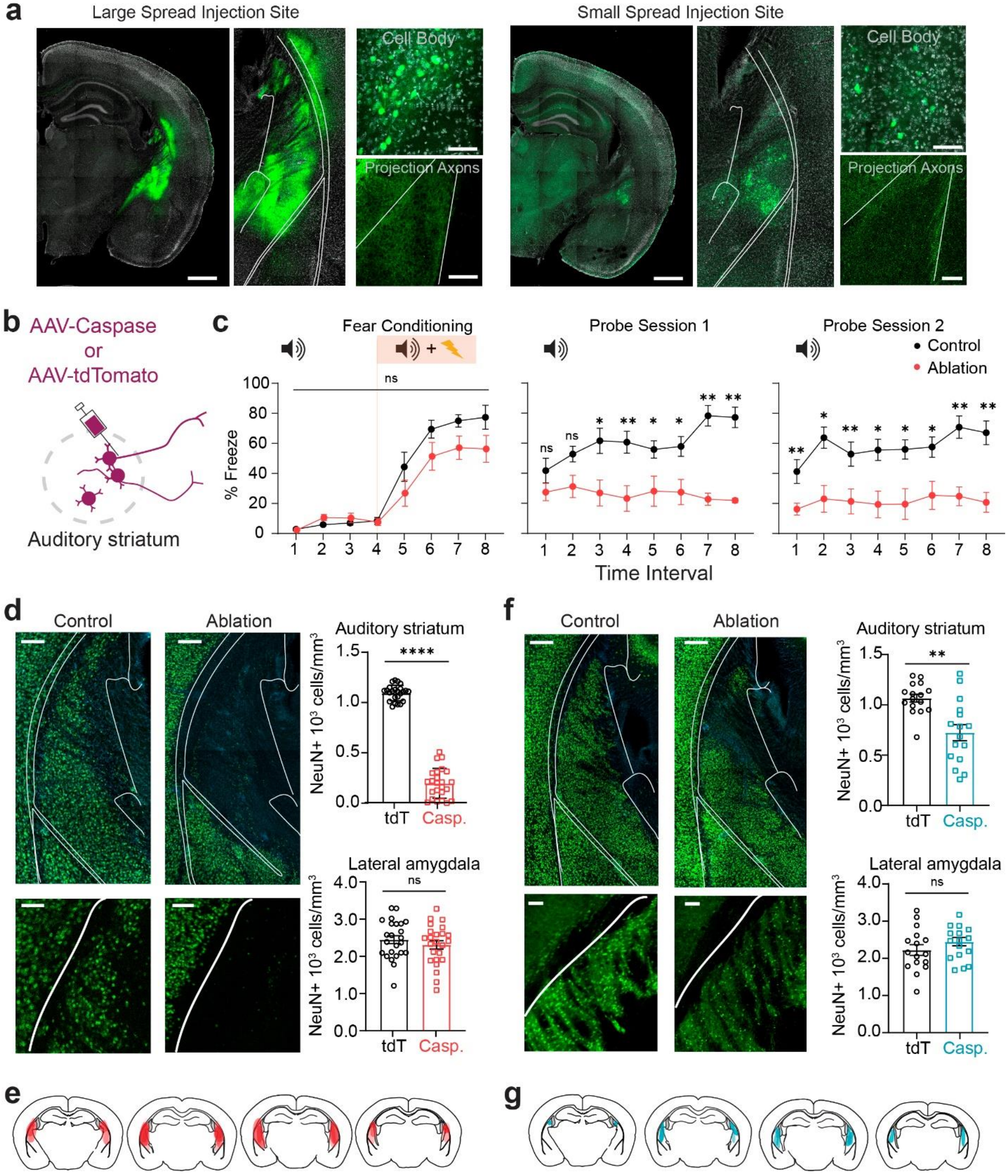

Supplementary Figure 2. Viral ablation of the auditory striatal neuronal population is spatially restricted. a, Two sets of anterograde tracing images exhibit the largest and smallest source spread when injecting a target volume of 250-300 nl tracing virus

(AAV9-eGFP) into the auditory striatum. Left, example of largest spread. Left is a 10X image of injection site; middle is zoom in image of injection site demonstrating auditory striatum and lateral amygdala border; right is a 40X image exhibiting cell bodies in the auditory striatum and axons in the lateral amygdala. Right, example of smallest spread. Left is a 10X image of injection site; middle is zoom in image of injection site demonstrating auditory striatum and lateral amygdala border; right is a 40X image exhibiting cell bodies in the auditory striatum and axons in the lateral amygdala. Scale bars are 400 µm for 10X injection site images and 100 µm for 40X images. **b**, Schematic for ablation of the auditory striatum without genetic restriction. **c**, Freezing behavior across cohorts of control vs. ablation. Freezing percentages for two consecutive tones were averaged as a single data point. n = 4 mice for each cohort. Error bars are SEM (two-sided unpaired Mann-Whitney test; ns $p > 0.05$, *$p < 0.05$; **$p < 0.01$). **d&e**, unrestricted ablation. **f&g**, projection-specific ablation. For **d&f**, n = 4 mice for each cohort. **d**, Images, auditory striatum in control animal with lateral amygdala shown, and comparable image with caspase ablation. The bottom two panels are respective zoomed-in images. Right top graph, Quantification of neuronal density in the auditory striatum between control and caspase groups for unrestricted ablation Control, 1.10 ± 0.02; Ablation, 0.20 ± 0.03 neuron x $10^3/mm^3$. Right bottom graph, quantification of neuronal density in the lateral amygdala between control and caspase groups for unrestricted ablation. Control, 2.45 ± 0.09; Ablation, 2.31 ± 0.10 neuron x $10^3/mm^3$. Error bars are SEM (two-sided unpaired Mann-Whitney test; ns p = 0.45 0.05, ****p = 0.00008). **e**, Schematic of viral spread and ablation spread for both control and ablation groups for experimental data shown in **d**. **f**, Histology of amygdala-projecting auditory

striatum neuronal ablation experiment performed in Figure 2. Images, auditory striatum in control animal with lateral amygdala shown, and comparable image with projection-specific caspase ablation. The bottom two panels are respective zoomed-in images. Right top graph, quantification of neuronal density in the auditory striatum between control and caspase groups for projection-specific ablation. Control, 1.07 ± 0.04; Ablation, 0.72 ± 0.08 neuron x $10^3/mm^3$. Right bottom graph, quantification of neuronal density in the lateral amygdala between control and caspase groups for projection-specific ablation experiments. Control 2.22 ± 0.18 neuron x $10^3/mm^3$; Ablation, 2.45 ± 0.11 neuron x $10^3/mm^3$. Error bars are SEM (two-sided unpaired Mann-Whitney test; ns $p = 0.80$, ****$p < 0.00002$). **g**, Schematic of viral spread and ablation spread for both control and ablation groups for experimental data shown in **f**. For histological images, labeled in blue is DAPI and green is the neuronal marker NeuN. Scale bars are 200 µm and 100 µm for overall and zoomed-in images, respectively. Source data are provided as a Source Data file.

Supplementary Figure 3

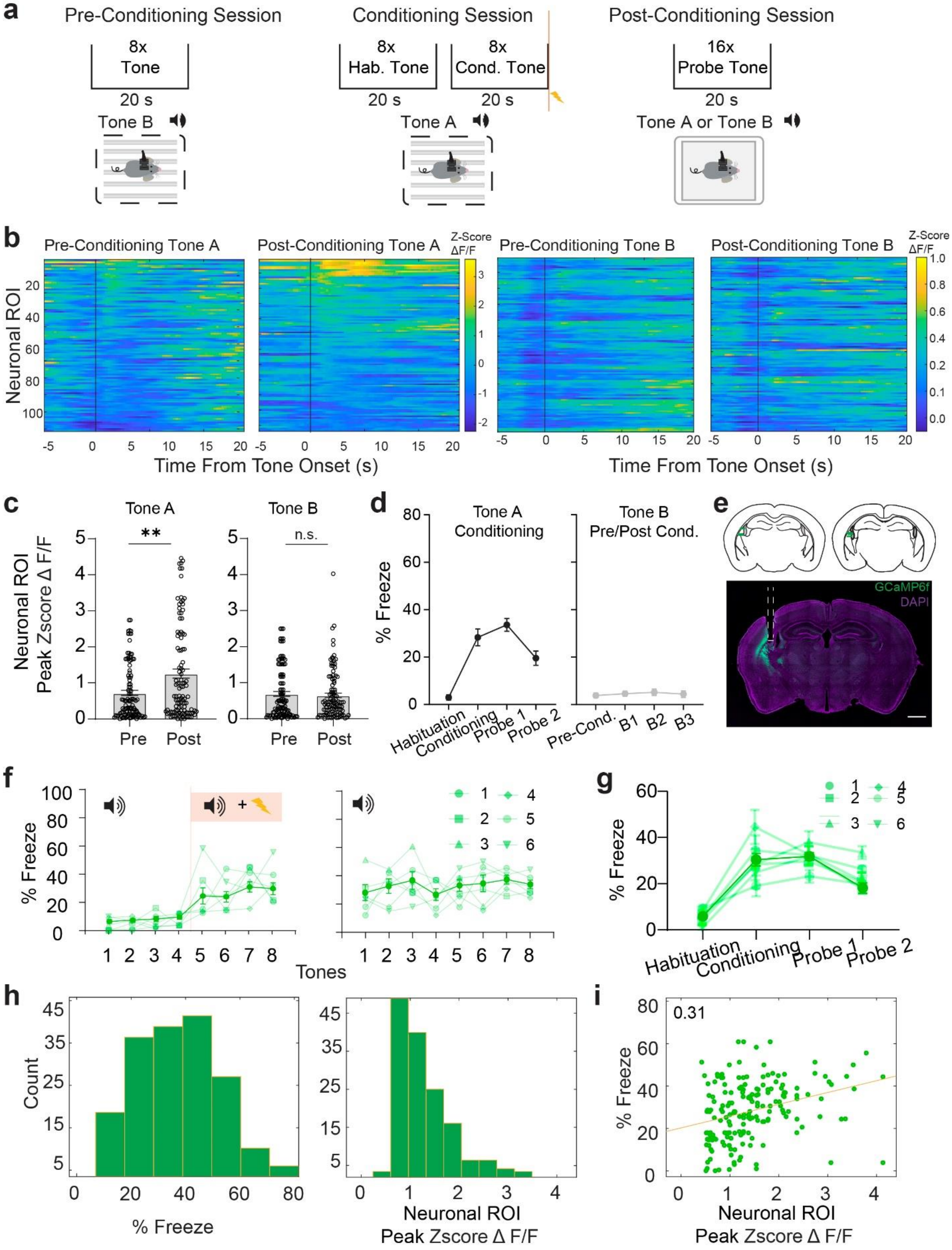

Supplementary Figure 3. Auditory striatal neurons show potentiated responses to paired tone but not non-paired tones and neuronal tonal responses are correlated with the freezing levels. **a**, Schematic for fear conditioning with Tone A paired with a shock and an innocuous Tone B. Eight 20-s, 10-kHz tones (Tone B) were presented to mice during a pre-conditioning session in a similar manner as in habituation. Subsequently, mice underwent auditory fear conditioning as in **Fig. 1** with 20-s Tone A. One day later, mice were again presented with Tone B in a different context. **b**, Heatmap showing neuronal ROI responses to Tone A (left two plots) and Tone B (right two plots) in pre- and post-conditioning sessions. **c**, Averaged neuronal activity in response to Tone A (left) and Tone B (right) during pre- and post-conditioning sessions. Error bars are SEM (n = 114 neuronal ROI across 4 mice; two-sided Wilcoxon rank-sum test; ns, p = 0.76; **p = 0.0092). **d**, Corresponding behavioral data for striatal neuronal imaging analysis. Left, averaged freezing percentage in response to tone A during habituation, conditioning, and probe sessions. Right, averaged freezing in response to tone B during pre-conditioning and post-conditioning sessions (3 post-conditioning tone B sessions; n = 4 mice). **e**, Left, example histology of lens placement and GCaMP6f expressed in the auditory striatum. Green, GCaMP6f; purple, DAPI. Scale bar = 1.0 mm. Right, summary of lens implantation sites. Each green line represents the bottom tip of a lens for each mouse. **f**, Freezing in response to tones during habituation, conditioning, and probe sessions. Freezing percentages for two consecutive tones were averaged as a single data point. Semi-transparent data points present each individual animal; each individual shape represents a different animal. Overall averaged data are stylized as solid. Error bars are standard error of the mean (SEM; n = 6 mice). **g**, Semi-

transparent data points present each individual animal; each individual shape represents a different animal. Overall averaged data are stylized as solid. Error bars are standard error of the mean (SEM; n = 6 mice). **h**, Distributions of freezing behaviors (left) and neuronal tonal responses (right) to individual tones in probe sessions. **i**, Correlation between the freezing behaviors and neuronal tonal responses. Pearson correlation, R = 0.3105; p = 1.88 x 10-5. n= 188 neurons from 6 mice. Source data are provided as a Source Data file.

Supplementary Figure 4

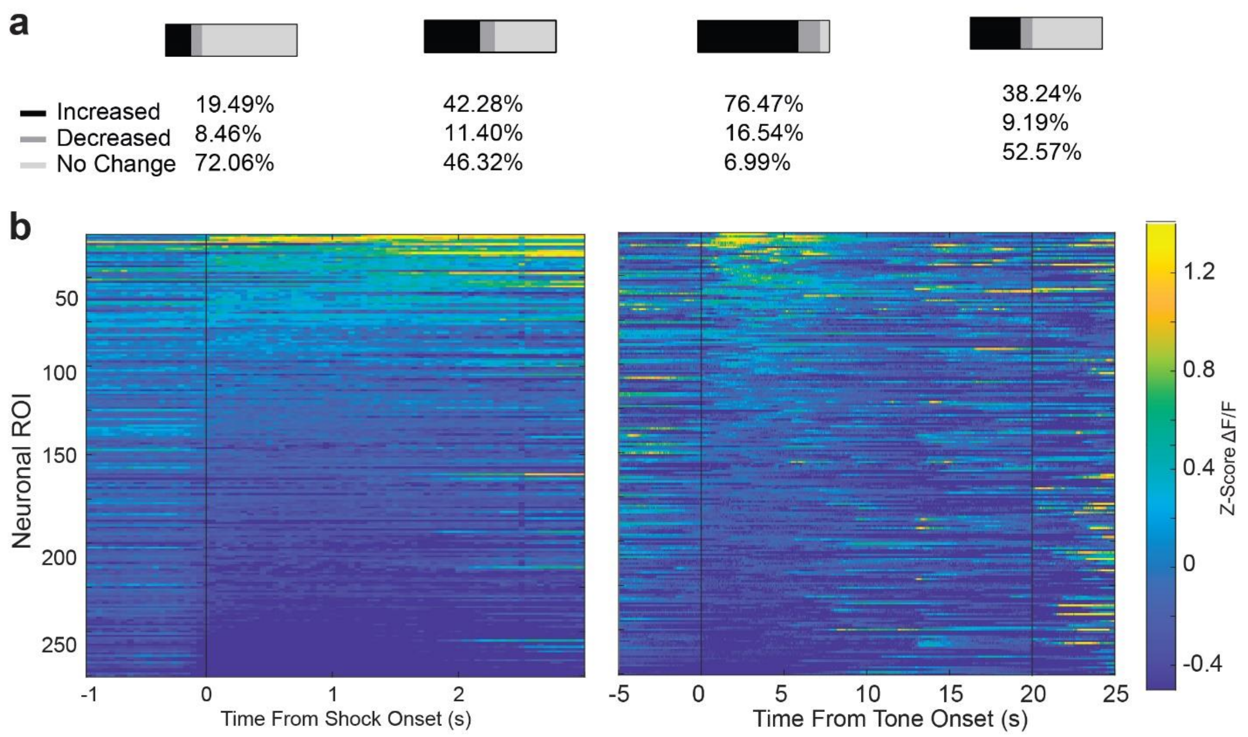

Supplementary Figure 4. The auditory striatal neuronal responses to conditioned tones are not linked with responses to the foot shocks. a, Using 5 s as the time window, the quantification of the proportion of neurons with increased (black), decreased (dark gray), or no significant change (light gray; Wilcoxon rank-sum test, $p > 0.05$) in response to tones. **b**, Left, heatmap of averaged ΔF/F neuronal responses towards foot shock. Right, the same neuronal ROIs arranged by tonal response amplitude with extended time course to include responses to foot shocks starting at the 20 s time point. n = 262 neuronal ROIs from 6 mice. Source data are provided as a Source Data file.

Supplementary Figure 5

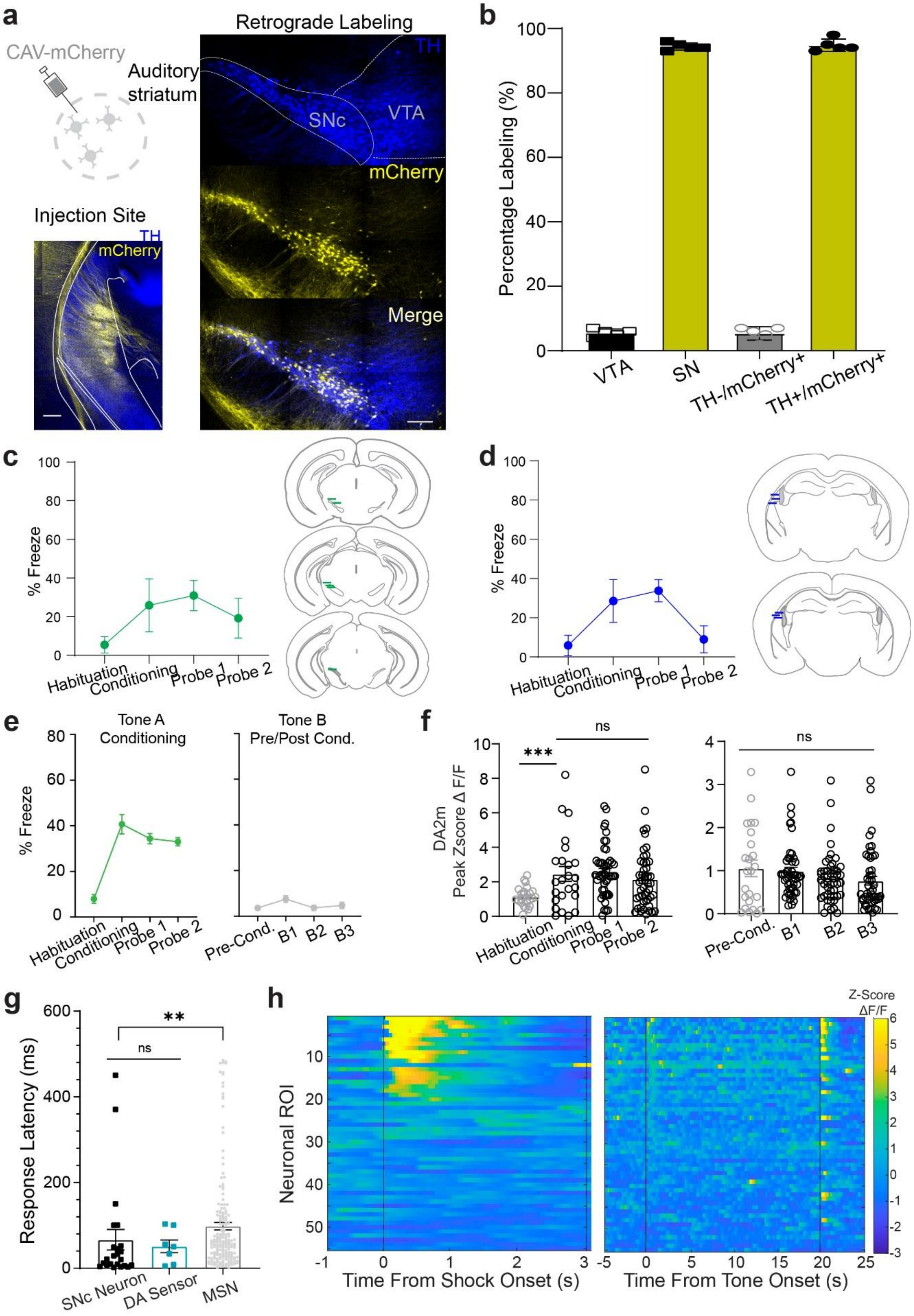

Supplementary Figure 5. SNc and DA imaging mice show freezing behavior after auditory fear conditioning. a, Left upper, injection schematic of CAV-mCherry into the auditory striatum. Left bottom, representative imaging of injection site and traversing mCherry+ axons in the auditory striatum. mCherry is pseudo-coloreed in gold, with TH labeling in blue. Scale bar is 100 µm. Right, retrograde labeling in midbrain dopamine regions, the VTA and SNc. **b**, Left two bars show percentage quantification of mCherry+ neurons in the VTA and SNc. Right two bars show the percentage of mCherry+ neurons that are either TH- or TH+ neurons. **c**, Averaged freezing percentage in response to tones during habituation conditioning, and probe sessions for SNc imaging mice. Summary of lens implantation sites for SNc imaging mice. Each green line represents the bottom tip of a lens for each mouse. n = 6 mice. **d**, Averaged freezing percentage in response to tones during habituation, conditioning, and probe sessions for dopamine sensor imaging mice. Summary of lens implantation sites for dopamine sensor auditory striatum imaging mice. Each blue line represents the bottom tip of a lens for each mouse. n = 6 mice. **e.** Corresponding behavioral data for striatal DA sensor mice. Left, averaged freezing percentage in response to Tone A (conditioned tone, 5 kHz) during habituation, conditioning, and probe sessions. Right, averaged freezing percentage in response to Tone B (unconditioned tone, 10 kHz) during pre-conditioning and post-conditioning sessions (n = 4 mice). **f.** Averaged and individual auditory striatal DA responses towards Tone A (left) or Tone B (right) during habituation, conditioning, and probe sessions. Individual dots represent averaged ΔF/F responses per two consecutive tones across three mice. Error bars are SEM (Wilcoxon rank-sum test; ns, p = 0.65; ***p = 0.0007; n = 3 mice). **g.** Comparison of tonal response latencies from SNc neuronal ROI

(n= 58 from 6 mice), DA sensor FOV (n = 6 mice), and auditory striatal neuronal ROI (n = 188 from 6 mice). Error bars are SEM (Mann-Whitney test; ns, $p = 0.18$; $^{**}p < 0.0073$). **h.** Left, heatmap of averaged ΔF/F SNc neuronal responses towards foot shock arranged by descending order of peak amplitude. Right, averaged ΔF/F SNc neuronal activity aligned to the onset of tones and arranged in descending order of tone response magnitude with time period extended to include shock period. Source data are provided as a Source Data file.

Supplementary Figure 6

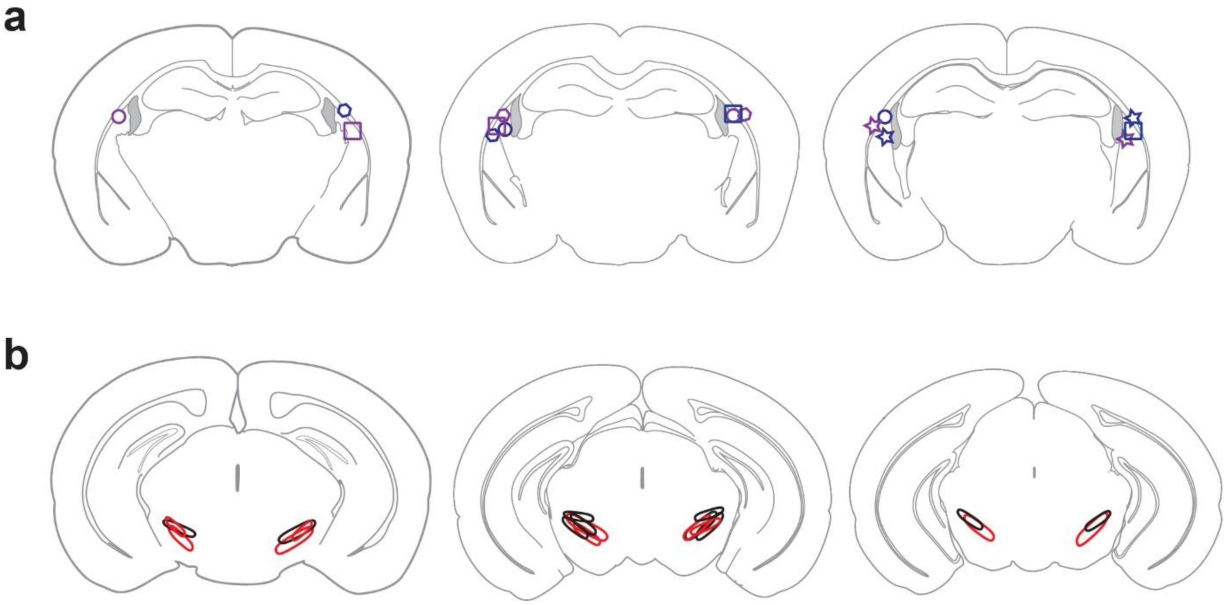

Supplementary Figure 6. Post hoc validation of nigrostriatal inhibition mice. a. Summary of optic fiber implantation sites for dopaminergic terminal inhibition for each mouse. Each symbol represents the bottom tip of an optic fiber for each mouse (purple, ArchT; blue, GFP. n = 4 mice for each group). The same symbol represents the bilateral sites from the same mouse. **b.** Summary of hM4Di (red) or mCherry (black) spread in chemogenetic inhibition mice. n = 5 mice for each group. Source data are provided as a Source Data file.
