# Supplementary material for: Nigrostriatal dopamine modulates the striatal-amygdala pathway in auditory fear conditioning
Source: Nat Commun. 2023 Nov 9;14:7231. doi: 10.1038/s41467-023-43066-9 (PMC10636191; doi:10.1038/s41467-023-43066-9)
Supplement: Supplementary file 3 — Reporting Summary [file 41467_2023_43066_MOESM3_ESM.pdf]

nature portfolio

Corresponding author(s): Qiaojie Xiong

Last updated by author(s): Oct 20, 2023

Reporting Summary

Nature Portfolio wishes to improve the reproducibility of the work that we publish. This form provides structure for consistency and transparency in reporting. For further information on Nature Portfolio policies, see our Editorial Policies and the Editorial Policy Checklist.

Statistics

For all statistical analyses, confirm that the following items are present in the figure legend, table legend, main text, or Methods section.

n/a	Confirmed	
[ ]	[x]	The exact sample size (*n*) for each experimental group/condition, given as a discrete number and unit of measurement
[ ]	[x]	A statement on whether measurements were taken from distinct samples or whether the same sample was measured repeatedly
[x]	[ ]	The statistical test(s) used AND whether they are one- or two-sided<br>*Only common tests should be described solely by name; describe more complex techniques in the Methods section.*
[ ]	[x]	A description of all covariates tested
[ ]	[x]	A description of any assumptions or corrections, such as tests of normality and adjustment for multiple comparisons
[ ]	[x]	A full description of the statistical parameters including central tendency (e.g. means) or other basic estimates (e.g. regression coefficient) AND variation (e.g. standard deviation) or associated estimates of uncertainty (e.g. confidence intervals)
[ ]	[x]	For null hypothesis testing, the test statistic (e.g. *F*, *t*, *r*) with confidence intervals, effect sizes, degrees of freedom and *P* value noted<br>*Give P values as exact values whenever suitable.*
[x]	[ ]	For Bayesian analysis, information on the choice of priors and Markov chain Monte Carlo settings
[x]	[ ]	For hierarchical and complex designs, identification of the appropriate level for tests and full reporting of outcomes
[ ]	[x]	Estimates of effect sizes (e.g. Cohen's *d*, Pearson's *r*), indicating how they were calculated

Our web collection on statistics for biologists contains articles on many of the points above.

Software and code

Policy information about availability of computer code

Data collection	Behavioral data were measured and acquired using custom-written scripts in MATLAB R2014a (The Mathworks, Inc., Natick, Massachusetts, USA) and via an automated video processing system (Freezeframe, Actimetrics). Data acquisition for microendoscopic recordings was performed using nVista Data Acquisition Software (Inscopix, Palo Alto, CA).
Data analysis	Behavioral data and associated parameters were analyzed using custom written scripts in MATLAB R2020b (The Mathworks, Inc., Natick, Massachusetts, USA). Microendoscopic data processing was performed using Mosaic (Inscopix, Palo Alto, CA). Cellular extraction and further data analysis was performed by using publicly available CNMF-E and custom written scripts in MATLAB. Statistical analyses were performed in Graphpad Prism 8 (Graphpad Software Inc.). Custom written codes for data acquisition and analysis are available upon request.

For manuscripts utilizing custom algorithms or software that are central to the research but not yet described in published literature, software must be made available to editors and reviewers. We strongly encourage code deposition in a community repository (e.g. GitHub). See the Nature Portfolio guidelines for submitting code & software for further information.

Data

Policy information about availability of data

All manuscripts must include a data availability statement. This statement should provide the following information, where applicable:

- Accession codes, unique identifiers, or web links for publicly available datasets
- A description of any restrictions on data availability
- For clinical datasets or third party data, please ensure that the statement adheres to our policy

All data are provided in the main text figures or supplementary data in the Source Data file. Source data are provided with this manuscript.

Research involving human participants, their data, or biological material

Policy information about studies with human participants or human data. See also policy information about sex, gender (identity/presentation), and sexual orientation and race, ethnicity and racism.

Reporting on sex and gender	N/A
Reporting on race, ethnicity, or other socially relevant groupings	N/A
Population characteristics	N/A
Recruitment	N/A
Ethics oversight	N/A

Note that full information on the approval of the study protocol must also be provided in the manuscript.

Field-specific reporting

Please select the one below that is the best fit for your research. If you are not sure, read the appropriate sections before making your selection.

[x] Life sciences [ ] Behavioural & social sciences [ ] Ecological, evolutionary & environmental sciences

For a reference copy of the document with all sections, see nature.com/documents/nr-reporting-summary-flat.pdf

Life sciences study design

All studies must disclose on these points even when the disclosure is negative.

Sample size	No statistical tests were used to pre-determine sample sized but the reported sample sizes are similar to what we and others have previously reported (Chen, et al., 2022, Shen et al., 2019; Zhong et al., 2019).
Data exclusions	For optogenetic, chemogenetic, and imaging data, animals were excluded if there were improper viral expression or implantation sites upon post-hoc inspection. No other animals were excluded for this study.
Replication	All details provided for methods, reagents, and data analysis were provided to ensure study repeatability. All sample sizes are reported in the main text, figure legends, and supplementary figure legends.
Randomization	Animals are randomly selected as experimental or control groups from the same litters. For experiments in auditory fear conditioning, animals were randomly allocated to control and experiemental groups prior to surgery.
Blinding	Experimenters handling animals in the behavioral fear conditioning paradigm were blinded to the allocation of control and experimental interventions. All behavioral and imaging data were collected using an automated fashion, and comparisons were drawn after time-stamp based analyses of data.

Reporting for specific materials, systems and methods

We require information from authors about some types of materials, experimental systems and methods used in many studies. Here, indicate whether each material, system or method listed is relevant to your study. If you are not sure if a list item applies to your research, read the appropriate section before selecting a response.

Materials & experimental systems

n/a	Involved in the study	
☐	☒	Antibodies
☒	☐	Eukaryotic cell lines
☒	☐	Palaeontology and archaeology
☐	☒	Animals and other organisms
☒	☐	Clinical data
☒	☐	Dual use research of concern
☒	☐	Plants

Methods

n/a	Involved in the study	
☒	☐	ChIP-seq
☒	☐	Flow cytometry
☒	☐	MRI-based neuroimaging

Antibodies

Antibodies used	The following primary antibodies and concentrations were used: goat anti-GFP (1:1000, Rockland 600-101-215), chicken anti-GFP (1:1000, Abcam ab13970), goat anti-RFP (1:1000, Rockland 200-101-379), rabbit anti-RFP (1:1000, Rockland 600-401-379), mouse anti-TH (1:1000, Millipore MAB5280), and rabbit anti-TH (1:1000; Abcam Ab112). The following secondary anti-bodies were used: donkey anti-chicken 488 (1:1000, Jackson ImmunoResearch 703-545-155), donkey anti-goat 488 (1:1000, ThermoFisher A-11055), donkey anti-rabbit 594 (1:1000, Jackson ImmunoResearch 711-587-003), donkey anti-rabbit 647 (1:1000, Jackson ImmunoResearch 711-605-152), donkey anti-mouse 594 (1:1000, Jackson ImmunoResearch 715-585-150), and donkey anti-mouse 647 (1:1000, Jackson ImmunoResearch 715-605-150).
Validation	All primary and secondary antibodies were validated in prior studies by our lab as well as others (2022 Chen et al., 2021 Liu et al., 2020 Wang et al., 2019 Chen et al., 2019 Shen et al.). For dopamine neuron identification, commonly used antibodies we used to label Tyrosine hydroxylase that have previously been used for both ex vivo and in vivo post-hoc analyses (2016 Parker et al.; 2018 da Silva et al.).

Animals and other research organisms

Policy information about studies involving animals; ARRIVE guidelines recommended for reporting animal research, and Sex and Gender in Research

Laboratory animals	C57BL/6J (The Jackson Laboratory) and DAT-IRES-Cre (The Jackson Laboratory, 006660) mice were used for this study. Both male and female 2-4-month-old mice were used. Mice were housed under similar conditions as previously reported (Chen et al., 2022; Liu et al., 2021) with 30-70% humidity, an ambient temperature of 64-97 oF, with free access to food and water, and under a 12-h light/dark cycle conditions.
Wild animals	This study did not involve wild animals.
Reporting on sex	Both male and female 2-4-month-old mice were used. Specific sex-based analyses were not performed with the observation of similar results between the two groups when addressing experimental questions approached and analyzed in order to draw conclusions for this study.
Field-collected samples	This study did not involve Field-Collected samples.
Ethics oversight	All animal procedures were approved by the Stony Brook University Animal Care and Use Committee and carried out in accordance with National Institutes of Health standards.

Note that full information on the approval of the study protocol must also be provided in the manuscript.

Plants

Seed stocks	N/A
Novel plant genotypes	N/A
Authentication	N/A
